# Data mining MR image features of select structures for lateralization of mesial temporal lobe epilepsy

**DOI:** 10.1371/journal.pone.0199137

**Published:** 2018-08-01

**Authors:** Fariborz Mahmoudi, Kost Elisevich, Hassan Bagher-Ebadian, Mohammad-Reza Nazem-Zadeh, Esmaeil Davoodi-Bojd, Jason M. Schwalb, Manpreet Kaur, Hamid Soltanian-Zadeh

**Affiliations:** 1 Radiology and Research Administration, Henry Ford Health System, Detroit, Michigan, United States of America; 2 Computer and IT Engineering Faculty, Islamic Azad University, Qazvin Branch, Qazvin, Iran; 3 Clinical Neurosciences Department, Spectrum Health Medical Group, Grand Rapids, Michigan, United States of America; 4 Physics Department, Oakland University, Rochester, Michigan, United States of America; 5 Neurosurgery Departments, Henry Ford Health System, Detroit, Michigan, United States of America; 6 CIPCE, School of Electrical and Computer Engineering, College of Engineering, University of Tehran, Tehran, Iran; McGill University, CANADA

## Abstract

**Purpose:**

This study systematically investigates the predictive power of volumetric imaging feature sets extracted from select neuroanatomical sites in lateralizing the epileptogenic focus in mesial temporal lobe epilepsy (mTLE) patients.

**Methods:**

A cohort of 68 unilateral mTLE patients who had achieved an Engel class I outcome postsurgically was studied retrospectively. The volumes of multiple brain structures were extracted from preoperative magnetic resonance (MR) images in each. The MR image data set consisted of 54 patients with imaging evidence for hippocampal sclerosis (HS-P) and 14 patients without (HS-N). Data mining techniques (i.e., feature extraction, feature selection, machine learning classifiers) were applied to provide measures of the relative contributions of structures and their correlations with one another. After removing redundant correlated structures, a minimum set of structures was determined as a marker for mTLE lateralization.

**Results:**

Using a logistic regression classifier, the volumes of both hippocampus and amygdala showed correct lateralization rates of 94.1%. This reflected about 11.7% improvement in accuracy relative to using hippocampal volume alone. The addition of thalamic volume increased the lateralization rate to 98.5%. This ternary-structural marker provided a 100% and 92.9% mTLE lateralization accuracy, respectively, for the HS-P and HS-N groups.

**Conclusions:**

The proposed tristructural MR imaging biomarker provides greater lateralization accuracy relative to single- and double-structural biomarkers and thus, may play a more effective role in the surgical decision-making process. Also, lateralization of the patients with insignificant atrophy of hippocampus by the proposed method supports the notion of associated structural changes involving the amygdala and thalamus.

## 1. Introduction

Hippocampal sclerosis is the most common abnormality observed in mesial temporal lobe epilepsy (mTLE) patients [1]. The salient features of hippocampal sclerosis on MR imaging are volume loss on T1-weighted imaging and signal hyperintensity on T2-weighted or fluid-attenuated inversion recovery (FLAIR) sequence. These features are seen in about 70% of cases [2] and when they are ipsilateral to the side of seizure onset seen on electroencephalographic (EEG) recordings, often lateralization of the epileptogenic side is assured [3, 4] permitting surgical resection of mesial temporal structures without need for further invasive studies [5–7]. However, some patients with mTLE have insufficient structural asymmetry on MRI when evaluated visually by experienced clinicians. Quantitative image analysis may detect structural asymmetry that is not obvious by visual inspection alone [6], although further challenges remain regarding lateralization accuracy in cases where hippocampal asymmetry is minimal or absent [4, 8–10]. Such analysis, when robust and concordant with scalp EEG and other clinical markers, may provide sufficient justification to avoid invasive electrographic monitoring and its risks [11].

Several MR image-based lateralization methods have concentrated on hippocampal attributes alone such as its volume [3, 12, 13] and signal intensity [14–16]. Recent studies have shown that structural volume loss is not limited to the hippocampus. The amygdala and parahippocampal gyrus can also be affected, and often changes may extend to extratemporal cortical regions and subcortical structures as well [17–19]. Some mTLE lateralization studies have analyzed neighboring structures, in an attempt to improve the accuracy of lateralization [4, 9, 10, 20–22]. Cendes et al [20] showed that combined volumetric features of both the hippocampus and amygdala resulted in a 92% lateralization accuracy concordant with EEG in a cohort of 31 mTLE patients. Keihaninejad et al [4] demonstrated that hippocampal and parahippocampal gyral volumes in mTLE patients with and without hippocampal sclerosis can lateralize mesial temporal epileptogenicity.

Although several multistructural lateralization studies [4, 9, 10, 20–22] have provided ample evidence of the utility of additional structural quantitative analysis, it is unclear whether there is an optimal limit to the number of such neuroanatomical sites that are needed to establish laterality. As increasing the number of neuroanatomical sites may lead to systematic errors and decreasing this number may limit the benefits of multistructural analysis, optimization is very important. By using data mining techniques [23], this study systematically weighs the influence of different neuroanatomical sites upon the determination of laterality and establishes a classifier that employs a minimum number of effective sites for lateralization to create a robust and reliable tool for mTLE cases.

## 2. Methods

This study investigates the contributions of select neuroanatomical regions toward the lateralization of mTLE in order to establish a minimum set of regions that demonstrate a greater predictability than other common hippocampal or multistructural volumetric approaches. In the proposed approach, the skull was stripped and neuroanatomical regions automatically segmented from T1-weighted MR images. Volumes of these defined regions were determined and normalized. A feature selection algorithm was applied to the extracted data to identify the most discriminative features. Subsets of these features were reviewed with a hill-climbing strategy in order to determine their roles in predicting the side of epileptogenicity. Logistic regression and the Support Vector Machine (SVM) method were also used to evaluate the performance of these markers for different classifiers. The rest of this section describes the approach in detail.

### 2.1 Patient population and MR imaging

In this study, we used MR images of 68 TLE patients. All material were de-identified based on a protocol that approved by IRB of Henry Ford Health System. Retrospective data from 68 unilateral mTLE patients, including 30 males (mean age 39.69±12.83) and 38 females (mean age 40.37±11.11), were analyzed. Table 1 shows clinical profiles of the patients who underwent a standard protocol of investigation that included inpatient scalp video-EEG, MRI, intracarotid amobarbital study and neuropsychological testing to establish their condition. Patients requiring extraoperative electrocorticography (eECoG) often underwent additional magnetoencephalography (MEG) and/or ictal and interictal single photon emission computed tomography (SPECT) or positron emission tomography (PET). The patients used in this study were all unilateral mTLE cases who had achieved an Engel class I outcome following surgical resection at Henry Ford Hospital between June 1993 and June 2009 and who also had acceptable FreeSurfer MRI segmentation results. The wide recruitment window allowed a sufficient number of patients to be accrued for the study and suitable follow-up, exceeding three years in all cases, was provided to declare a genuine outcome. Surgery consisted of an inferior temporopolar topectomy with amygdalohippocampectomy. Resections were performed on the left side in 39 patients and on the right in 29. Of the 68 cases, 28 (41%) required extraoperative electrocorticography (eECoG). The MR image characteristics of the patients were qualitatively evaluated by neuroradiologists who identified the presence of hippocampal sclerosis (HS-P) in 54 cases or its absence in the remaining 14 cases (HS-N). Their evaluations were based on hippocampal volume loss on T1-weighted images and signal hyperintensity on FLAIR images. Although pathological study of the excised tissue is not relevant to the decision-making process entailed in surgical candidacy, it is included here as a correlative feature of interest. This revealed either a qualitatively mild hippocampal sclerosis or a focal sclerosis in 3 of the 14 HS-N cases. Among the 54 HS-P cases, 15 were qualitatively assessed to have the characteristically stringent features of hippocampal sclerosis (HS) while a further 19 cases demonstrated a predominant gliosis with variable cell loss that was judged to be less notable. Among 50 HS-P cases that had undergone histopathological study, only five were judged normal in appearance and were likely the result of sampling as the entire hippocampus was often not extracted. Moreover, pathological expression throughout the hippocampus is not uniform as might be expected from MR imaging [16]. Gliosis itself is responsible for the hyper intensity seen on T2 weighted MR imaging [24, 25] so that there may be some variation in the interpretation of MTS among a number of neuroradiologists. Ultimately, in the context of this study, it is the neuroimaging that must be scrutinized as the preoperative measure of concern in order to establish its worth as a qualifying metric.

**Table 1:** Clinical profiles of patients. Patients are identified by sex, race, handedness, seizure class, duration of epilepsy, age at surgery, side of surgery, the need for intracranial electrographic study (II) in addition to the preliminary scalp EEG study (I) and follow-up period after surgery. Histopathology is indicated, when available. The presence or absence of mesial temporal sclerosis (MTS) according to neuro-radiological report establishes the preoperative qualitative interpretation.

No.	Sex	Race	Hnd	Seizure Class	Epilepsy Duration (y)	Age at Surgery (y)	Side	EEG	Pathology	MTS
1	F	W	R	CP	25	59	L	I	CD, GL	N
2	F	A	R	CP	14	30	R	I,II	GL	N
3	M	W	R	CP	7	27	R	I	ND	N
4	F	W	R	CP	39	53	L	I, II	HS	N
5	F	W	R	CP	23	30	L	I, II	GL	N
6	F	W	L	CP	15	38	R	I, II	NA	N
7	F	W	R	CP	22	39	R	I, II	ND	N
8	F	W	R	CP	12	48	L	I	FS	N
9	F	W	R	CP	24	25	L	I, II	HS	N
10	M	W	R	CP	9	43	R	I, II	ND	N
11	F	W	R	CP	31	33	R	I, II	NL	N
12	M	W	R	CP	46	56	L	I	NL	N
13	F	W	R	CP	29	45	R	I, II	ND	N
14	F	W	R	CP	4	47	L	I, II	NL	N
15	M	W	L	SP	59	61	L	I	HS	Y
16	F	NA	R	SP	36	56	L	I	GL	Y
17	M	W	L	CP	1	38	L	I, II	HS	Y
18	F	B	R	CP	19	37	R	I	FS	Y
19	M	NA	Amb	CP	34.5	36	R	I	GL	Y
20	M	W	R	CP	58.5	60	L	I	HS	Y
21	M	W	R	CP	40	48	R	I	ND	Y
22	M	W	L	CP	18	30	L	I	FS	Y
23	M	AI	R	CP	20.5	24	L	I	ND	Y
24	F	W	R	CP	44	55	L	I	HS	Y
25	F	W	R	CP	27	28	R	I	NL	Y
26	F	W	R	CP	17	48	R	I	FS	Y
27	M	W	R	CP	6	21	L	I, II	ND	Y
28	F	W	R	CP	18.4	20	L	I, II	NA	Y
29	M	W	R	CP	49	51	R	I, II	ND	Y
30	F	B	R	CP	37	49	L	I, II	FS	Y
31	F	W	R	CP	13	64	L	I, II	HS	Y
32	M	W	R	CP	25	30	L	I, II	NA	Y
33	F	W	L	CP	20	37	L	I, II	ND	Y
34	M	W	R	CP	33	56	R	I	GL	Y
35	F	W	R	CP	40	42	L	I, II	GL	Y
36	M	W	L	CP	29	31	L	I, II	GL	Y
37	F	W	R	CP	24	34	R	I	ND	Y
38	M	W	R	CP	47	47	L	I	ND	Y
39	F	W	L	CP	22	50	R	I	GL	Y
40	F	W	R	CP	35	38	R	I	GL	Y
41	M	W	R	CP	17	19	L	I	NL	Y
42	F	W	R	CP	20	31	R	I	GL	Y
43	M	B	R	CP	30	39	L	I	HS	Y
44	F	W	L	CP	9	28	L	I	GL	Y
45	M	W	R	CP	33	34	R	I, II	FS	Y
46	F	W	R	CP	26	34	R	I	ND	Y
47	F	W	R	CP	34	44	R	I	GL	Y
48	F	W	R	CP	10	23	R	I, II	GL	Y
49	M	W	R	CP	15	52	L	I	GL	Y
50	F	W	R	CP	NA	48	L	I	GL	Y
51	M	W	R	CP	6	41	R	I	NL	Y
52	M	W	R	CP	20	25	R	I	NA	Y
53	F	W	R	CP	19	39	L	I	NA	Y
54	F	NA	R	CP	45	45	L	I, II	GL	Y
55	M	W	R	CP	22	32	R	I	ND	Y
56	F	W	R	CP	18	24	L	I	NL	Y
57	M	NA	R	CP	9	27	R	I	GL	Y
58	F	W	R	CP	10	14	R	I, II	GL	Y
59	M	W	R	CP	20	53	R	I, II	GL	Y
60	F	A	L	CP	30	51	L	I	HS	Y
61	F	W	R	CP	47	48	R	I	GL	Y
62	M	W	R	CP	2	20	L	I, II	NL	Y
63	F	W	R	CP	18	38	L	I	GL	Y
64	M	W	R	CP	27	28	L	I	CD	Y
65	M	W	R	CP	28	30	L	I	ND	Y
66	M	W	R	CP	33	53	L	I, II	HS	Y
67	F	NA	R	CP	44	43	L	I	HS	Y
68	M	W	R	CP	5	45	L	I, II	HS	Y

**Abbreviations**: A: Asian, AI: American Indian, Amb: Ambidexterity, B: Black, CD: Cortical Dysplasia, CP: Complex Partial, F: Female, FS: Focal Sclerosis, GL: Gliosis, Hnd: Handedness, HS: Hippocampal Sclerosis, L: Left, M: Male, MTS: Mesial Temporal Sclerosis, N: No, NA: Not available, ND: Non-diagnostic, NL: Normal, R: Right, SP: Simple Partial, W: White, Y: Yes, y: Year

There were two reasons for the inclusion of the HS-P patients in this study: 1) although the hippocampal volume change is obvious for this group, volume changes of other neuroanatomical sites and the extent of these changes in mTLE lateralization were unclear; and, 2) as HS-N patients appear to constitute only 30% of mTLE patients [2], the sample size for HS-N cases was relatively small, increasing the chance of overfitting in the machine learning process and limiting generalization of the extracted rules.

The data of 68 mTLE patients included 39 left and 29 right laterality. The mean of epilepsy duration for two groups of left and right were respectively 25.93±15.10 and 23.60±11.58 years. These values for age at surgery were respectively 40.64±13.17 and 37.48±10.02 years. To statistically evaluate the group differences in terms of epilepsy duration and age at surgery variables, we applied a two-sample assuming unequal variances t-test on each variable. The p-values of the t-tests for epilepsy duration and age at surgery variables were respectively 0.477 and 0.265, which were greater than the significance level of 0.05, confirming no statistically significant differences between the means of the corresponding groups.

Preoperative coronal T1-weighted MR images were acquired using inversion recovery spoiled gradient echo (IRSPGR protocol) on a 1.5T or a 3.0T MRI system (Signa, GE, Milwaukee, USA). For the 1.5T MRI, the imaging parameters were: TR/TI/TE = 7.6/1.7/500 ms, flip angle = 20°, voxel size = 0.781 mm × 0.781 mm × 2.0 mm, matrix size = 256×192, and FOV = 220 mm × 220 mm. For the 3.0T MRI, the imaging parameters were: TR/TI/TE = 10.4/4.5/300 ms, flip angle = 15◦, voxel size = 0.39 mm × 0.39 mm × 2.0 mm, matrix size = 320 × 192, and FOV = 200 mm × 200 mm. In all cases, the SNR was above 80. Materials for generating T2 mapping were available for 40 cases (33 HS-P and 7 HS-N) in our institution. Dual echo imaging protocol using echo times (TE) of 30 and 90 ms and repetition time of 2.5 sec was employed to acquire axial images covering the whole brain with the slice thickness of 5 mm, 2.5 mm gapping, FOV of 200×200 mm^2^, and the resulting image size of 256×256. T2 maps were estimated by fitting a single exponential decay function to the data. For each subject, the T2-weighted image with smaller TE value was registered to the T1-weighted image using the coarse registration method implemented in SPM8 (http://www.fil.ion.ucl.ac.uk/spm/). Then, the resulting transformation was used to align the T2 map to the T1-weighted image for further processing.

### 2.2 Brain segmentation and feature extraction

**Fig 1:**
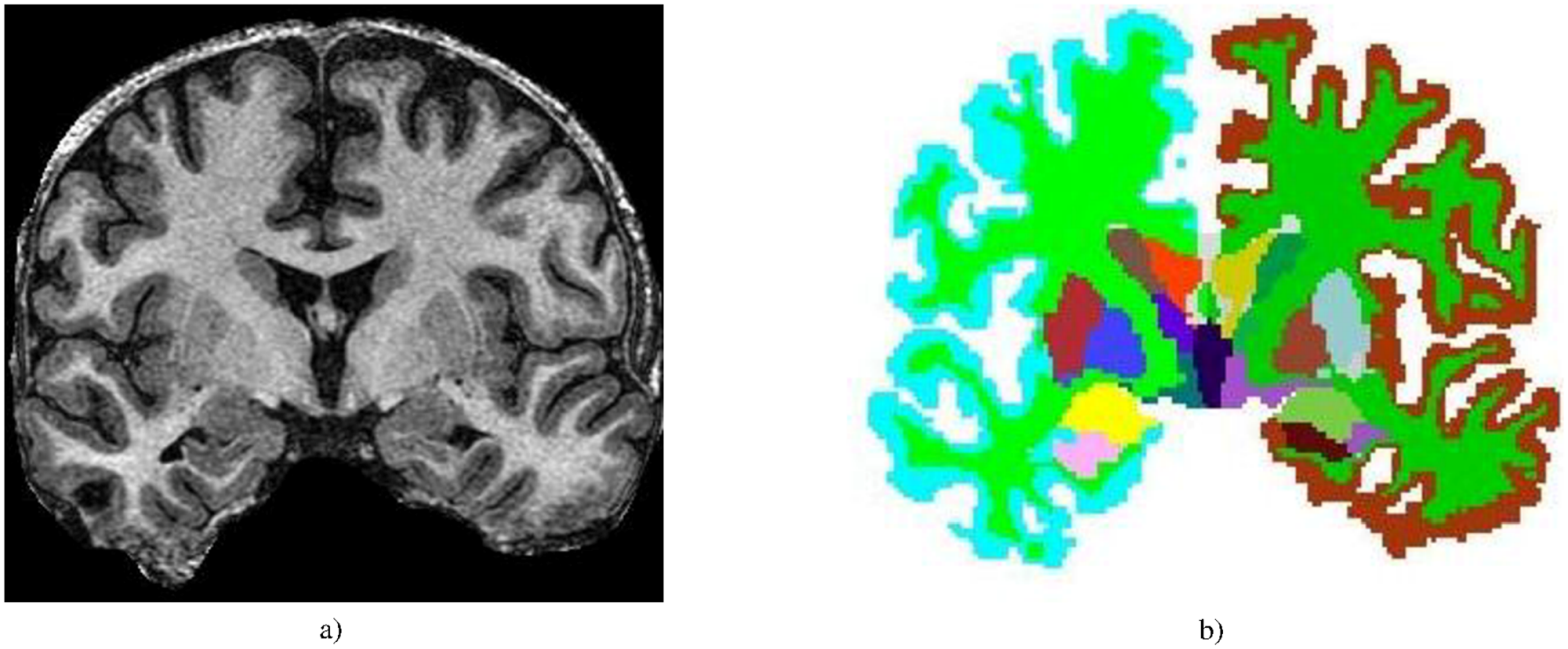
a) A coronal T1-weighted brain MRI. b) Brain structures segmented by FreeSurfer.

Automatic segmentation of paired structures in each cerebral hemisphere (Fig 1) was performed using FreeSurfer software [26] (version 5.3.0) under Linux Debian 6.0.5 release. The volumetric features were extracted from the segmented structures using an in-house code written in MATLAB and WEKA (Waikato Environment for Knowledge Analysis) [27]. Table 2 provides a list of all interhemispherically paired structures that were delineated by the subcortical segmentation and cortical parcellation modules of FreeSurfer. The volume difference of each pair of structures was normalized to their summation and multiplied by 100 to show the percentage of the relative volume change:

$$f_{i}=\frac{v_{Li}-v_{Ri}}{v_{Li}+v_{Ri}}*100\;i=1,\;\ldots ,\;53$$ (1)

where *v*_*Li*_ and *v*_*Ri*_ are the left and right volumes, respectively, of the i^th^ structure. We consider *f*_*i*_ as a feature for each pair of structures demonstrating a normalized volume difference of the right structure relative to the left.

**Table 2:** List of segmented hemispherically-paired neuroanatomical regions by FreeSurfer.

1. Cerebral Exterior	2. Cerebral White Matter	3. Cerebral Cortex	4. Lateral Ventricle	5. Inferior lateral Ventricle	6. Cerebellum Exterior
7. Cerebellum White Matter	8. Cerebellum Cortex	9. Thalamus	10. Caudate	11. Putamen	12. Pallidum
13. Hippocampus	14. Amygdala	15. Nucleus accumbens	16. Substantia Nigra	17. Ventral Diencephalon	18. Vessel
19. Choroid plexus	20. Banks superior temporal sulcus	21. Caudal anterior cingulate cortex	22. Caudal middle frontal gyrus	23. Cuneus cortex	24. Entorhinal cortex
25. Fusiform gyrus	26. Inferior parietal cortex	27. Inferior temporal gyrus	28. Isthmus-cingulate cortex	29. Lateral occipital cortex	30. Lateral orbital frontal cortex
31. Lingual gyrus	32. Medial orbital frontal cortex	33. Middle temporal gyrus	34. Para-hippocampal gyrus	35. Para-central lobule	36. Pars opercularis
37. Pars orbitalis	38. Pars triangularis	39. Pericalcarine cortex	40. Post-central gyrus	41. Posterior-cingulate cortex	42. Precentral gyrus
43. Precuneus cortex	44. Rostral anterior cingulate cortex	45. Rostral middle frontal gyrus	46. Superior frontal gyrus	47. Superior parietal cortex	48. Superior temporal gyrus
49. Supramarginal gyrus	50. Frontal pole	51. Temporal pole	52. Transverse temporal cortex	53. Insula volume	

### 2.3 Feature selection

Cell death from recurrent excitation results in atrophy of hippocampal and parahippocampal structures. Pathophysiological extension to extratemporal cortical and subcortical structures transynaptically [17–19], may affect related sites in a similar fashion. Classifier performance may decrease with the addition of correlated structures; therefore, correlated features should be removed from the training set.

To eliminate the negative impact of both correlated and irrelevant features (i.e., structures) in the decision-making process, a feature selection stage is required. A wrapper subset evaluator [28] was used to establish an optimal subset of features that generated the highest classification accuracy for lateralization. This approach employed a supervised learning algorithm to evaluate different subsets of features. Since the search space is large and demands an extensive search time, the wrapper algorithm expedited the process by identifying a suboptimal feature set in a reasonable time. This algorithm worked on the basis of a hill-climbing strategy and selected structures step-by-step with greater information content for mTLE lateralization.

### 2.4 Determination of epileptogenic side

Upon selection of an optimal set of structures, training of a learning machine proceeded using retrospective training data. In this study, two different supervised classifiers, logistic regression and support vector machine, were used and their results compared. These two classifiers are described below.

#### Logistic regression

Logistic regression is a popular and robust supervised classifier widely used in biostatistics [29]. In this study, in order to avoid overfitting, a multinomial logistic regression with a ridge estimator [30] was used. As shown in Eq (2), the kernel of the multinomial model function computed the probability of each class. In this equation, *j* is the number of the current class, *k* is the total number of classes, *f*_1_..*f*_*m*_ are features and $\beta_{0}^{j}..\beta_{m}^{j}$ denote the coefficients that are optimized by the classification algorithm during the training phase.

$$Pr[j|f_{1},f_{2},\ldots ,f_{m}]=\frac{e^{\beta_{0}^{j}+\beta_{1}^{j}f_{1}+\beta_{2}^{j}f_{2}+\cdots +\beta_{m}^{j}f_{m}}}{1+\sum_{j=1}^{k-1}e^{\beta_{0}^{j}+\beta_{1}^{j}f_{1}+\beta_{2}^{j}f_{2}+\cdots +\beta_{m}^{j}f_{m}}}$$ (2)

#### Support vector machine

**Fig 2:**
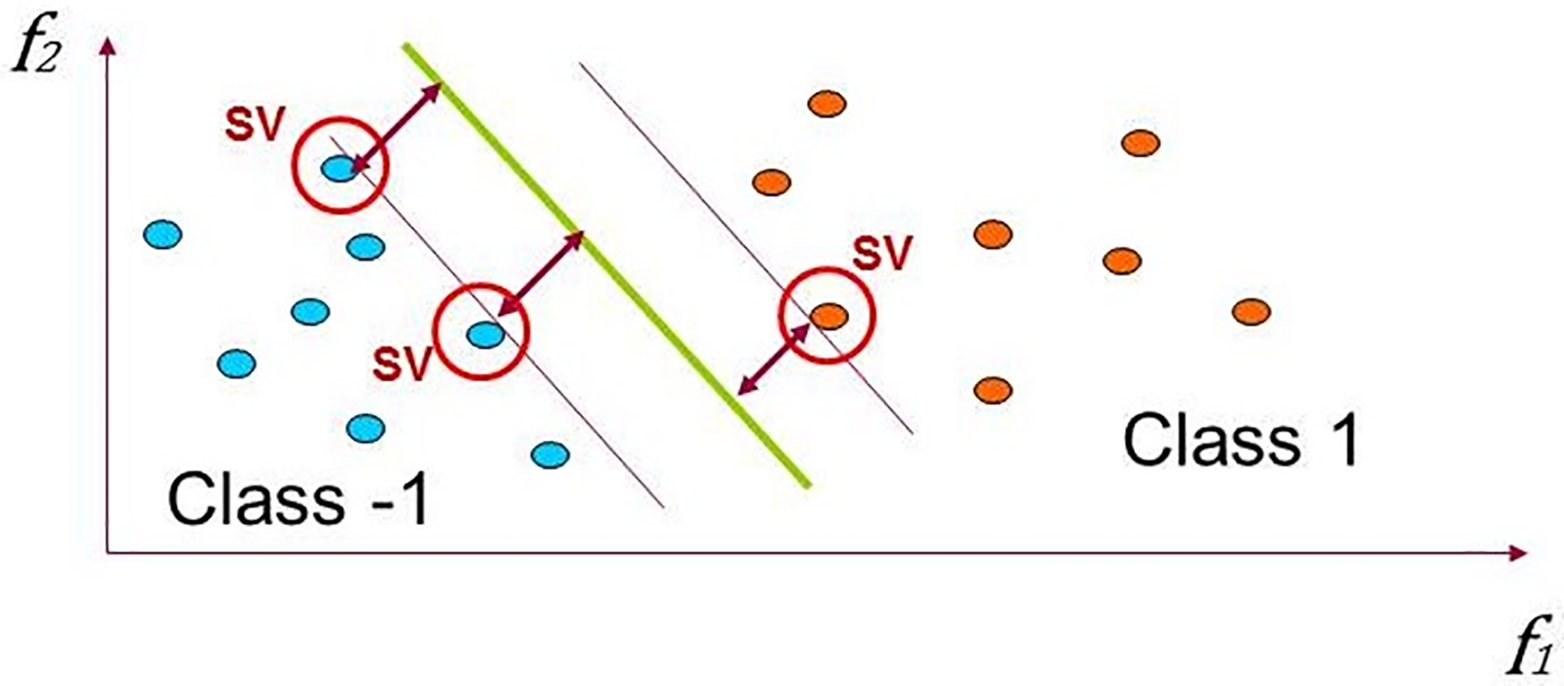
Linear discrimination, maximizing the margin in SVM.

The support vector machine (SVM) is a special kind of linear model called the maximum-margin hyperplane. Eq (3) shows the general form of a hyperplane in the m-dimensional space as a function of features and weight coefficients. Here *f*_1_..*f*_*m*_ are features and *w*_1_..*w*_*m*_ are coefficients that SVM tunes based on the training data. Fig 2 visualizes how the SVM tunes the coefficients and forms a maximum-margin hyperplane in a two-dimensional space with a two-class dataset that are linearly separable. At first, the SVM discovers a small number of critical boundary instances (i.e., support vectors) in the training set. Then, by tuning the *w* coefficients, it builds a linear discriminant function that separates support vectors as widely as possible.

$$F(w,f)=w_{0}+w_{1}f_{1}+w_{2}f_{2}+\cdots +w_{m}f_{m}$$ (3)

In this study, *SPegasos* was used as an efficient version of linear support vector machines. It applies a stochastic subgradient descent algorithm for optimizing *w* coefficients [31].

## 3. Results

### 3.1 Feature selection

The wrapper algorithm with logistic regression (i.e., the learner machine) was used to find the best subset. The input feature set consisted of 53 volumetric features of the structure pairs listed in Table 2 and segmented by FreeSurfer. The data was divided into 10 folds and feature selection was done separately for each fold. The volumetric features selected most in each fold defined the best subset demonstrating good lateralization performance. These included the hippocampus, amygdala, thalamus, putamen, cerebral white matter, entorhinal cortex, inferior temporal gyrus, paracentral lobule, postcentral gyrus and parahippocampal gyrus. These 10 structures were considered as a set of promising features and refined further in the classification phase.

### 3.2 Classification using various subsets of features

In order to establish whether the 10 selected features shared dependency, a hill-climbing strategy was used to find an optimal subset. Table 3 presents the classification results for the logistic regression classifier using a leave-one-out cross-validation evaluation. In order to avoid overfitting, this strategy was applied in all of the subsequent experiments. As a first step of the hill-climbing strategy, the hippocampus was found to be the best single marker for mTLE lateralization.

**Table 3:** Effect of quantitative volumetry of different structures on the accuracy of mTLE lateralization.

1st Step	H ^1^	A ^2^	T ^3^	P ^4^	CWM ^5^	EC ^6^	ITG ^7^	PCL ^8^	PCG ^9^	PHG ^10^
	**82.4%**	73.5%	57.4%	55.9%	77.9%	64.7%	61.8%	57.4%	57.4%	72.0%
2nd Step	H+A	H+T	H+P	H+CWM	H+EC	H+ITG	H+PCL	H+PCG	H+PHG	
	**94.1%**	82.4%	80.9%	85.3%	82.4%	83.8%	80.9%	82.4%	82.4%	
3rd Step	H+A+T	H+A+P	H+A+CWM	H+A+EC	H+A+ ITG	H+A+ PCL	H+A+ PCG	H+A+ PHG		
	**98.5%**	94.1%	94.1%	92.6%	94.1%	91.2%	92.6%	91.2%		
4th Step	H+A+T+P	H+A+T+CWM	H+A+T+EC	H+A+T+ITG	H+A+T+PCL	H+A+T+PCG	H+A+T+PHG			
	**98.5%**	97.1%	92.6%	95.6%	95.6%	95.6%	92.6%			
5th Step	H+A+T+P+CWM	H+A+T+P+EC	H+A+T+P+ITG	H+A+T+P+PCL	H+A+T+P+PCG	H+A+T+P+PHG				
	**97.1%**	95.6%	92.6%	94.1%	94.1%	91.2%				

^1^ Hippocampus

^2^ Amygdala

^3^ Thalamus

^4^ Putamen

^5^ Cerebral White Matter

^6^ Entorhinal Cortex

^7^ Inferior Temporal Gyrus

^8^ Para-Central Lobule

^9^ Post-Central Gyrus

^10^ Para-Hippocampal Gyrus

**Fig 3:**
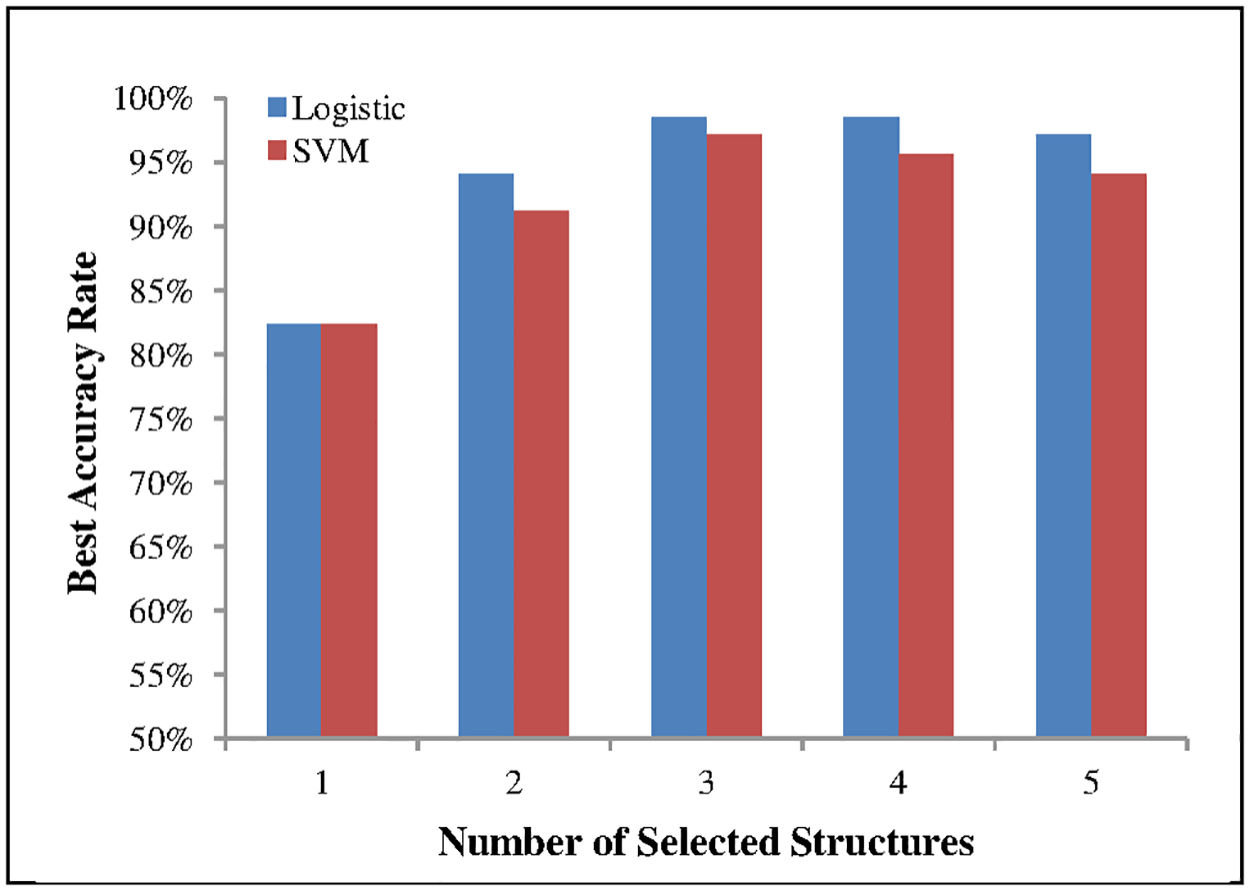
Best accuracy as a function of the number of selected structures.

With the initial structure now established, the hill-climbing strategy was applied to the remaining structures sequentially. Sets of two features for mTLE lateralization were evaluated at a time. The second step showed the hippocampus and amygdala predominate as the best two structures for mTLE lateralization. In the third step, sets of three structures were examined with the results demonstrated in Table 3. Logistic regression generated the most accurate lateralization results using the hippocampus, amygdala and thalamus. The fourth and fifth steps evaluated sets of four and five structures, respectively. Fig 3 summarizes the results presented in the five steps. This diagram shows that the set of the three former structures provided optimal accuracy for lateralization without further reliance upon the remaining structures.

### 3.3 Support vector machine

To evaluate the reliability of the selected features, experiments were repeated with a support vector machine (SVM) as a linear classifier and the results compared with those found by logistic regression (Fig 3). Although the logistic regression showed slightly superior performance, the SVM results appeared very similar demonstrating that the selected structures bore sufficient stability. Fig 3 shows that the proposed ternary-structural volumetric biomarker is independent of the classifier in that the changing pattern of accuracy as a function of the number of structures selected is the same for the two classifiers used in the study.

### 3.4 Single-structure lateralization

**Fig 4:**
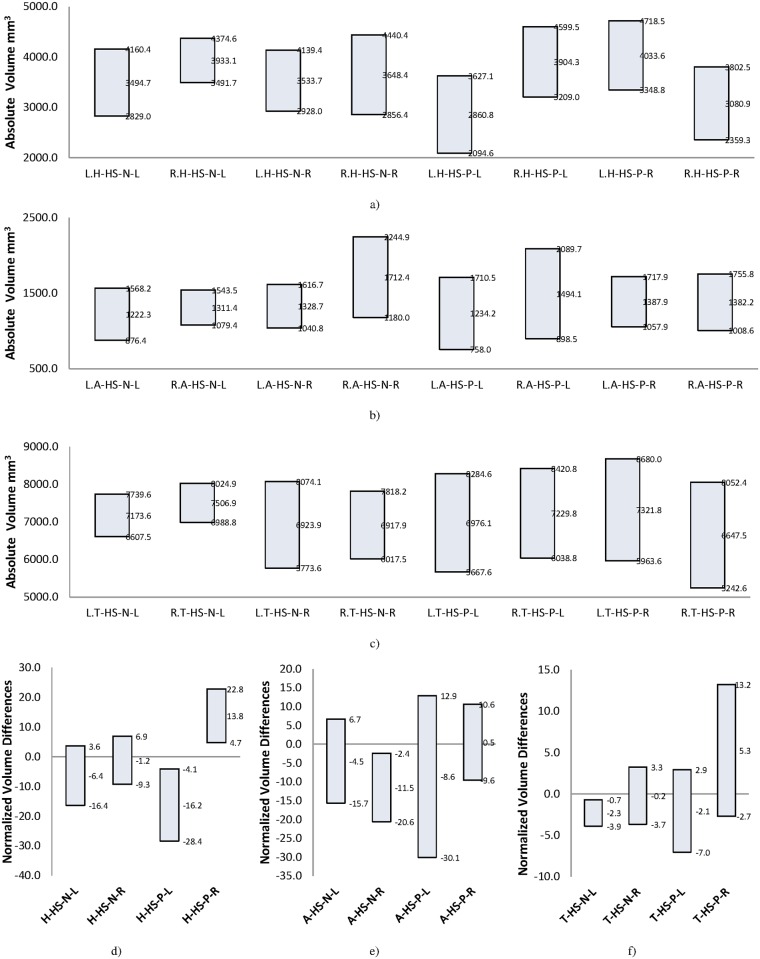
Mean ± SD range for volume of different structures in HS-P and HS-N groups. a) Absolute volume of hippocampus. b) Absolute volume of amygdala. c) Absolute volume of thalamus. d) Normalized volume differences (atrophy) of hippocampus. e) Normalized volume differences of amygdala. f) Normalized volume differences of thalamus. Bar label pattern for a, b, and c is W.X-HS-Y-Z and for d, e, and f is X-HS-Y-Z where, W is the side of structure (L/R means left/right), X is the brain structure (H/A/T means Hippocampus/Amygdala/Thalamus), HS-Y identifies the group of patients (HS-N/HS-P) and Z is the side of epileptogenicity (L/R means left/right). For example: L.T-HS-P-R is Left Thalamus volume of cases with Hippocampal Sclerosis and Right side of epileptogenicity.

In order to assess the utility of each of the three selected structures as a solitary marker of laterality, both the mean and standard deviation for each structure were evaluated individually. Fig 4(a)–4(c) shows the mean and standard deviation ranges of absolute volumes of the three structures: hippocampus, amygdala and thalamus. These were categorized based on the individual structure, mTLE group and side of epileptogenicity. The absolute volume for each structure was found to have sufficient overlap in both HS-N and HS-P groups to disqualify it as a suitable marker for lateralization purposes. Fig 4(d)–4(f) shows the same values for normalized volume differences (i.e., atrophy) based on Eq (1). Fig 4d shows that this normalized feature for the hippocampus has no overlap in the HS-P group. In clinically relevant terms, as an individual marker, atrophy of the hippocampus is sufficient for mTLE lateralization in the HS-P group. Fig 4(e) and 4(f) shows atrophy of other structures (i.e., amygdala and thalamus) but neither one is sufficient as a single structural marker for mTLE lateralization in either of the HS-N and HS-P groups.

### 3.5 Multistructural lateralization

**Fig 5:**
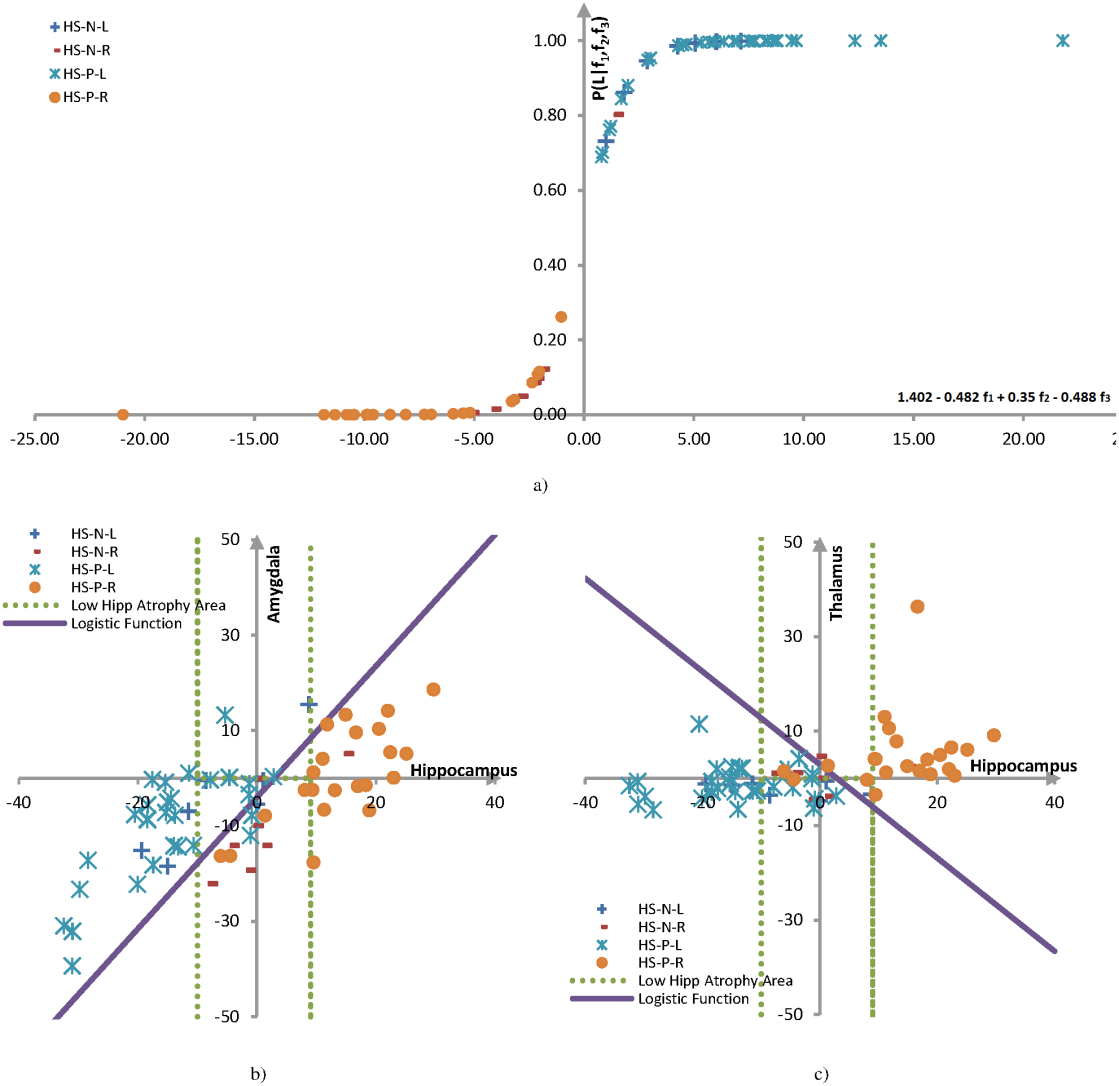
Analysis of the logistic regression for decision-making using the proposed three-structure marker. a) Probability of lateralization to the left for all mTLE cases based on Eq (4). b) Projection of logistic decision boundary for mTLE lateralization samples onto the amount of atrophy on the hippocampus-amygdala space. c) Projection of logistic decision boundary for mTLE lateralization samples onto the amount of atrophy on the hippocampus-thalamus space.

In order to establish the proposed three-structure marker for mTLE lateralization as a sufficient discriminator, the probability of lateralization was computed for the logistic regression. As the number of features was reduced to three following the feature selection phase, the general form of the logistic probability function of Eq (2) was simplified to:

$$Pr[L|f_{1},f_{2},f_{3}]=\frac{e^{\beta_{0}^{L}+\beta_{1}^{L}f_{1}+\beta_{2}^{L}f_{2}+\beta_{3}^{L}f_{3}}}{1+e^{\beta_{0}^{L}+\beta_{1}^{L}f_{1}+\beta_{2}^{L}f_{2}+\beta_{3}^{L}f_{3}}}$$ (4)

where, ***f***_**1**_, ***f***_**2**_, ***f***_**3**_ are the respectively normalized volumetric features (i.e., atrophies) for the hippocampus, amygdala and thalamus and the values of the regression parameters, $\beta_{0}^{L}$, $\beta_{1}^{L}$, $\beta_{2}^{L}$ and $\beta_{3}^{L}$ are, respectively, 1.402, -0.482, 0.350 and -0.488. The values were computed based on 1*10^−8^ for the ridge parameter. Fig 5a illustrates the probability of lateralization for all 68 mTLE patients. The proposed three-structure marker classifies all cases correctly except for one HS-N case. Table 4 shows performance for mTLE lateralization in detail for the proposed biomarker, evaluated by the leave-one-out and 5-folds cross validation methods. Accuracy, true left and right rates are calculated based on Eqs (5)–(7) below, where *TL*, *FL*, *TR* and *FR* are, respectively, the number of true left, false left, true right and false right samples assigned by the classifier. As the 5-folds cross-validation approach uses a smaller training set relative to the leave-one-out methodology, its learning power is less than the latter. Consequently, the performance measures of the 5-fold method are smaller than those of the leave one out method.

**Table 4:** Results of the proposed three-structure volumetric marker for HS-P and HS-N groups.

Patient Groups	Number of Samples	Leave one out			5-folds cross validation		
		Accuracy Rate	True Left Rate	True Right Rate	Accuracy Rate	True Left Rate	True Right Rate
HS-P	54	100%	100%	100%	94.4%	90.6%	100%
HS-N	14	92.9%	87.5%	100%	85.7%	85.7%	85.7%
Total	68	98.5%	97.5%	100%	92.6%	89.7%	96.6%

AccuracyRate=(TL+TR)/(TL+FL+TR+FR) (5)

TrueLeftRate=TL/(TL+FR) (6)

TrueRightRate=TR/(TR+FL) (7)

In order to illustrate the discriminating role of each structure in mTLE lateralization, a decision boundary domain was computed for the logistic regression. Assuming a probability of 0.5 corresponds to the decision boundary, the decision boundary is determined as follows:

$$Pr[L|f_{1},f_{2},f_{3}]=0.5$$ (8)

$$e^{\beta_{0}^{L}+\beta_{1}^{L}f_{1}+\beta_{2}^{L}f_{2}+\beta_{3}^{L}f_{3}}=0.5+0.5(e^{\beta_{0}^{L}+\beta_{1}^{L}f_{1}+\beta_{2}^{L}f_{2}+\beta_{3}^{L}f_{3}})$$ (9)

$$e^{\beta_{0}^{L}+\beta_{1}^{L}f_{1}+\beta_{2}^{L}f_{2}+\beta_{3}^{L}f_{3}}=1$$ (10)

$$\beta_{0}^{L}+\beta_{1}^{L}f_{1}+\beta_{2}^{L}f_{2}+\beta_{3}^{L}f_{3}=0$$ (11)

Eq (11) specifies a plane in the 3-dimentional feature space. In order to generate a practical demonstration, the projection of this plane is drawn in two 2D spaces. Fig 5(b) and 5(c) shows the logistic decision boundary, respectively, in the hippocampus-amygdala and hippocampus-thalamus spaces. All HS-P and HS-N cases are also shown in these spaces. The diagrams distinguish the cases with hippocampi showing distinct volume differences, including most of the HS-P cases, from the rest. In other words, the cases that appear outside the dotted lines can be easily lateralized by the hippocampal feature individually. The cases without qualitative volumetric differences, including most HS-N patients, appear within the dotted area and are lateralized by their amygdalar and thalamic features.

## 4. Discussion

A new and more expedient tristructural imaging biomarker is proposed for the lateralization of mesial temporal epileptogenicity, based upon an analysis of normalized volumes of neuroanatomical sites within and outside of the limbic system. The goal was to identify a minimum set of structures that, when considered together, would reliably predict laterality and outperform hippocampal or other multistructural options. Combined hippocampal and amygdalar volume analysis correctly lateralized 94.1% of the cases compared to only 82.4% when hippocampal volumes were assessed solely in a cohort of patients manifesting a unilateral TLE. With the addition of the corresponding thalamic volume, correct lateralization was achieved in 98.5% of cases, a 4.4% improvement relative to that attained with analysis of hippocampal and amygdalar volumes. The tristructural metric correctly lateralized the epileptogenic side in all cases with a demonstrated hippocampal sclerosis and in 92.9% of those without, supporting the notion of an associated structural change involving both the amygdala and thalamus.

### 4.1 Overfitting avoidance

Overfitting avoidance is one of the main purposes of any adaptive modeling study. Actually, there is a trade-off between underfitting and overfitting when the size of the training and testing datasets are limited. In the present study, using four separate subsets of the data for training and testing (with 68/4 = 17 samples in each subset) for feature selection and classification phases may reduce overfitting but causes underfitting and severely impacts the learning power of the model. In order to keep a balance between underfitting and overfitting, cross-validation was performed in all studies reported in this paper. However, different folding parameters were used in different experiments to control the bias; 10-fold for feature selection and 5-fold and leave-one-out for classification. This procedure is attractive for two reasons. First, the greatest possible amount of data is used for training, which presumably increases the generalization of the results and accuracy of the classifier. Secondly, the use of different folding parameters and averaging of the test results ensure that the results are not achieved by chance. A multinomial logistic regression with a ridge estimator was also used for the same reason. Le Cessie and Van Houwelingen [30] showed that ridge estimators could improve parameter estimation and reduce prediction error with small population sizes.

### 4.2 FreeSurfer segmentation

The quality of image segmentation has a significant impact upon the extracted features and the training of the classifiers. The FreeSurfer software is widely used as a segmentation tool [32–34] although some studies have identified systematic errors. Using the Dice coefficient, Akhondi-Asl et al [13] showed that hippocampal volumes extracted by FreeSurfer did differ from that obtained by manual segmentation. Germeyan et al [35] applied FreeSurfer on a mixed set of 1.5T and 3T images and demonstrated that hippocampal volumes extracted by FreeSurfer software were larger than manually segmented hippocampi in both epileptic patients and nonepileptic subjects. Hippocampal volume ratios (i.e., right:left), however, were not altered significantly by the segmentation method. Keller et al [36] demonstrated that thalamic volume extraction by a manual stereological approach was in agreement with that identified by FreeSurfer software.

The present study did not take into account any systematic error relevant to segmentation that was inherent in the FreeSurfer software. Visual inspection of the segmented structures allowed exclusion of the low quality images from the study. However, no absolute measures of structural volumes were used to ensure consistency for comparison of cases. The data of the 68 mTLE patients used in the study included 42 1.5T and 26 3T images. Using 5-folds cross-validation, there were 3 and 2 wrong classification samples for 1.5T and 3T subsets, respectively. In other words, accuracy rates for the proposed classifier were 92.9% and 92.3% for the 1.5T and 3T subsets, respectively. These close accuracy rates confirm that the end result of the proposed methods does not depend on the field strength.

### 4.3 Single modality approaches

Single modality MR imaging for mTLE has been shown to achieve limited accuracy in the lateralization of epileptogenicity. Jafari-Khouzani et al [16] used hippocampal fluid-attenuated inversion recovery (FLAIR) MR imaging in 25 nonepileptic control subjects and 36 mTLE patients. Image intensity, represented by mean and standard deviation, was determined for the hippocampal region and a boundary domain established to distinguish results obtained from control subjects. A lateralization accuracy of 75% was declared for all cases identified lying outside the boundary domain. A similar approach was used with subtraction single photon emission computed tomography (SPECT) imaging where SPECT images were coregistered to MRI and a lateralization accuracy of 89% was achieved [22]. Kerr et al [37] developed an automated computer-aided diagnostic (CAD) tool for localizing the epileptogenic focus in mTLE using interictal fluorodeoxyglucose positron emission tomography (FDG-PET; iPET). Using long term video-EEG monitoring outcomes as their only standard of laterality, the accuracy rate ranged from 76% to 89% with different confidence intervals. Nazem-Zadeh, et al [38] investigated the lateralization capability of diffusion parameters in 20 mTLE cases that had undergone surgery and obtained Engel class I outcomes. Using an uncertainty analysis approach, they found that the mean diffusivity (MD) of the hippocampus and the fractional anisotropy (FA) of the posteroinferior cingulum and crus of the fornix could lateralize 18, 15 and 14 of the 20 cases, respectively. Within this limited population, the lateralization accuracy of these biomarkers was 90%, 75% and 70%, respectively. Shishegar, et al [39] studied shape features of the hippocampus for the lateralization of mTLE patients. They used the Laplace Beltrami operator and spherical harmonics to extract shape features and support vector machine (SVM) classifiers to lateralize their cases. On a database of 59 mTLE patients, they achieved 86% and 85% accuracy rates for the Laplace Beltrami operator and the spherical harmonics methods, respectively. These results demonstrate the limitations of single modality models for mTLE lateralization.

### 4.4 Multimodality approaches

Several studies have employed multimodality models for mTLE lateralization to better inform the decision-making process. Zhang et al [40] reviewed 24 mTLE patients with and without hippocampal sclerosis, some manifesting a bilateral temporal epileptogenicity. Presurgical evaluation consisted of MRI, MR proton spectroscopy (^1^H-MRS), video-EEG with some patients requiring further intracranial EEG study (i.e., eECoG). For patients with evident hippocampal sclerosis, MRI and ^1^H-MRS showed a high (100%) concordant lateralization in cases of unilateral mTLE, whereas, in the case of patients without hippocampal sclerosis, ^1^H-MRS showed moderate (i.e., 60–75%) concordance. Although a multimodal approach, consisting of EEG, MRI, MRS and PET or SPECT, was suggested as a means of further distinguishing laterality in the more difficult cases, no quantitative results were presented in support. Nazem-Zadeh et al [41] applied a multimodal response model to determine mTLE laterality using T1-weighted MRI volumes, mean and standard deviation FLAIR intensity and the means of normalized ictal-interictal SPECT intensity of the hippocampi in 45 mTLE cases which had achieved an Engel class I outcome. These were compared to a cohort of 20 control, nonepileptic subjects. A 100% lateralization accuracy was achieved although no indication was given regarding the presence or absence of hippocampal sclerosis. Kim et al [42] proposed a multispectral and multimodal approach based on high-resolution T1- and T2-weighted MRI with hippocampal subfield segmentations, to carry out lateralization efforts in mTLE patients. Fifteen mTLE patients with normal hippocampal volumes were studied and the proposed approach correctly lateralized all. Coan et al [43] used both quantitative hippocampal volume and T2 relaxometry to aid in defining hippocampal sclerosis. Their volumetry results showed 95% and 13% accuracy rates respectively on HS-P (125 cases) and HS-N (78 cases) groups. After adding T2 values, they achieved 99% and 28% accuracy rates respectively on HS-P and HS-N groups, showing 4% and 15% improvements.

As multimodal approaches used in the investigation of TLE appear to improve upon the reliability of determining laterality when compared to single modality approaches, we investigated possible improvement by adding T2 relaxometry results to the present study similar to Coan et al [43] mentioned above. Materials for generating T2 mapping were available for 40 of the cases (33 HS-P and 7 HS-N). T2 map asymmetry analysis for the hippocampal region showed 84.8% and 85.7% lateralization accuracy for the HS-P and HS-N groups, respectively, but this information did not improve the 100% and 93% accuracy rates achieved by our multistructural technique.

### 4.5 Multistructural approaches

Multistructural approaches, similar to multimodal approaches, offer the same reliability in prediction but at reduced cost and risk to the patient. Barron et al [44] proposed a multistructural biomarker based on the functional connection strength among four structures: thalamus, hippocampus, entorhinal cortex and amygdala. They predicted the seizure onset zone with an 86% sensitivity and 100% specificity in 24 mTLE patients. Their lateralization accuracy, albeit with a smaller cohort and involving four sites, is comparable to that of the current study. In another study, a similar multistructural volumetric approach was undertaken by Keihaninejad et al [4]. However, a comparison with the current model shows that a reduction in the number of chosen neuroanatomical sites expedites the analysis over that proposed by Keihaninejad et al [4]. In those cases in which a hippocampal sclerosis was manifest, similarly robust outcomes were demonstrated with four sites compared to three in our study. For those cases without sclerosis, the current model proved 8% more accurate (93% vs 85%) with only three structures contrasted to their 17 structures.

## 5. Conclusion and future work

The findings of the present study may increase efficacy of lateralization using MR imaging alone in the treatment of drug-resistant mTLE patients. The greater accuracy and convenience of the proposed tristructural MR imaging biomarker in determining laterality of ictal onset of mTLE patients, relative to the conventional method of hippocampal analysis, makes it attractive for epilepsy surgery decision-making. In the absence of definitive volumetric hippocampal asymmetry, this approach makes use of associated changes in the epileptogenic network, specifically the amygdala and thalamus, to provide a higher lateralization accuracy. We plan to extend the proposed method to lateralize mTLE patients based on the multimodal imaging data prospectively and to further increase accuracy and confidence in the surgery decision-making process.
