# Correction: Data mining MR image features of select structures for lateralization of mesial temporal lobe epilepsy

**DOI:** 10.1371/journal.pone.0209866

**Published:** 2018-12-20

**Authors:** Fariborz Mahmoudi, Kost Elisevich, Hassan Bagher-Ebadian, Mohammad-Reza Nazem-Zadeh, Esmaeil Davoodi-Bojd, Jason M. Schwalb, Manpreet Kaur, Hamid Soltanian-Zadeh

There are errors in the affiliations for author Hassan Bagher-Ebadian. The correct affiliations should be: **4** Physics Department, Oakland University, Rochester, Michigan, USA, **7** Radiation Oncology, Henry Ford Health System, Detroit, Michigan, USA.
